# Editorial: Host-microbe interaction in SARS-CoV-2 infection: mechanism and intervention

**DOI:** 10.3389/fimmu.2023.1198868

**Published:** 2023-04-18

**Authors:** Arnaud John Kombe Kombe, Tengchuan Jin

**Affiliations:** ^1^ Department of Obstetrics and Gynecology, The First Affiliated Hospital of University of Science and Technology of China (USTC), Division of Life Sciences and Medicine, University of Science and Technology of China, Hefei, Anhui, China; ^2^ Laboratory of Structural Immunology, Chinese Academy of Sciences (C.A.S.) Key Laboratory of Innate Immunity and Chronic Disease, Division of Life Sciences and Medicine, University of Science and Technology of China, Hefei, Anhui, China; ^3^ Healthy Processed Foods Research Unit, United State Department of Agriculture, Agriculture Research Service (USDA-ARS), Western Regional Research Center, Albany, CA, United States; ^4^ CAS Center for Excellence in Molecular Cell Science, Chinese Academy of Science, Shanghai, China

**Keywords:** COVID-19, SARS-CoV-2, immune response, virus-host interaction, intervention, pathophysiology, sepsis

Since December 2019, the world has experienced a nightmare due to the new Severe Acute Respiratory Syndrome Coronavirus 2 (SARS-CoV-2) causing the deadliest coronavirus disease known as COVID-19. This severe disease is characterized by metabolic acidosis, acute respiratory distress syndrome (ARDS), septic shock, and multiple organ dysfunction. Lacking specific treatment to contain the rapid infection spread and mitigate the disease burden, we have accumulated knowledge to understand the virus biology, its interactions with host cells, undelaying mechanisms of infection, the pathophysiology, and the immune response to the virus. However, because of the complexity of these aspects, especially the molecular interactions between SARS-CoV-2 and the host immune system, which are crucial for the successful therapeutic design, our knowledge is still limited, rendering the pandemic management challenging, talk less of the evolutionary nature of SARS-CoV-2.

This Research Topic aimed to gather novel findings and up-to-date conclusive studies from multidisciplinary expertise regarding the uncovered aspects of SARS-CoV-2–host cell interaction, which we believe, might bring light to developing effective prophylaxis and therapeutic interventions. This collection of 13 insightful studies can be put along a continuum to illustrate the immunological and molecular mechanisms underlying the COVID-19 severity, the progression of COVID-19 from the early stage board that features the later severity of the infection establishing the diverse immunological markers, and, therefore, the alternative means proposed to control the disease.

One of the major concerns (if not the first) in COVID-19 fighting strategies is the continuous emergence of new SARS-CoV-2 variants, especially the variants of concern (VOCs) with their ability to escape the immune response. Omicron, for instance, the last known heavily mutated SARS-CoV-2 VOC, plays a crucial role in COVID-19 severity as it can escape natural and vaccine-induced immune response, and because of its high transmissibility. In their studies, Shah and Woo and Wang et al. demonstrated through and confirmed once again, at the molecular and clinical levels, the mechanism underlying the immune escape by Omicron variants, precisely the most evolved Omicron sub-strains BA.1, BA1.1, and BA.2. As previously reviewed (1) and among the over 30 mutations carried by Omicron variants, Shah and Woo demonstrated that mutations T478K, Q493K, Q498R, and E484A significantly contribute to the approved therapeutic antibody (etesevimab, bamlanivimab, and CT-p59) escape and fast transmissibility of Omicron by dampening antibody neutralizing effects and substantial enhancement of ACE2 binding affinity of RBD. More worrying, these studies showed that, as for REGEN-COV (casirivimab + imdevimab) inefficacy against Omicron (2), Omicron mutations significantly reduce neutralizing efficacy of bivalent antibody cocktails, including regdanvimab (etesevimab + CT-p59), and AZ combo (COV2-2196 + COV2-2130) targeting different SARS-CoV-2 epitopes, idea which was first thought as a better alternative to mitigate transmission and prevent the emergence of new variants (1).

Similarly, the contributive study of Tang et al. in this issue supports the before-mentioned concluding studies. In a vesicular stomatitis virus (VSV)-based pseudovirus infection system, Tang et al. showed that the infectivity of the Delta variant (B.1.617.2) critically increased, compared to that of previously evolved variants, including the D6114G variant, with the ability to resist neutralizing/inhibiting effects of RBD- and NTD-targeting antibodies and vaccinated sera, explaining its high transmissibility and severity. Therefore, even though BRII-196 + BRII-198 could retain protective effects (Wang et al.), more potent combinations of neutralizing antibodies from more than two groups (1) should be designed, or other alternatives need to be developed for more effective therapy against Omicron and Delta variants (talk less of other VOCs), potential upcoming emerging variants, and in case of infection with multiple SARS-CoV-2 variants. Saying the latter, protease inhibitors, which still can hamper entry and protect against SARS-CoV-2 infection regardless of mutational rate in spike protein [Tang et al. (3)], might be regarded as an ideal alternative.

Besides new variants, secondary bacterial coinfections can worsen primary SARS-CoV-2 infection, leading to severity. It was previously demonstrated that patients infected with respiratory tract bacteria are susceptible to SARS-CoV-2, worsening COVID-19 severity to fatality (4). The study by Smith et al. further demonstrated from the COVID-19 mice model that primary SARS-CoV-2 infection also increases susceptibility and aggravation to other respiratory infections, including pneumonia. Specifically, while the SARS-CoV-2 viral load remains steady (and low to a less extent) during the infection course, the bacterial load was progressively enhanced and accompanied by a progressive increase of neutrophils, pulmonary bacterial-associated burden, and bacteremia, which feature susceptibility to and severity of secondary bacterial infections during SARS-CoV-2. A low level of pulmonary macrophage required to clear bacteria-infected cells, which could be caused by INF response-inducing SARS-CoV-2, might be an underlying mechanism describing the pathophysiology of the coinfection. This study constitutes a point of alarm as, despite COVID-19 vaccines that reduce the severity of SARS-CoV-2 infection and thus the associated fatalities on the one hand and the observed reduced transmission of many pathogens on the other hand, asymptomatic or mild COVID-19 patients are on risk for bacterial pneumonia complication. Therefore, while encouraging COVID-19 vaccination, pneumonia diagnostic should be established in COVID-19 patients (and *vis-versa*), and adequate antibiotic treatment should be started in confirmed bacteria-co-infected patients.

The previous collection of studies highlighted new molecular and immunological aspects of the implication of SARS-CoV-2 VOCs and the pathogenic pool in the severity of COVID-19. Furthermore, several other studies have been carried out to assess the fate (mild, moderate, or severe) of a COVID-19 infection (prognostic) from the early stage (3-5 days post-symptom onset) (5, 6). Knowing the initial immune response in the early phase of SARS-CoV-2 infection and its effects on the development of respiratory failure is essential for quickly taking action to prevent fatalities. Xu et al. demonstrated that a transient high IFN-I response alongside a delayed adaptive immunity at the early stage of the infection is a hallmark of severe COVID-19. Specifically, they found that the early stage of COVID-19 severity is characterized by a strong INF response, which then drops rapidly throughout the infection in severe COVID-19 patients. In contrast, in mild COVID-19, the early INF response was low and stood steady.

Moreover, analyses starting from the early stage of severe COVID-19 showed that myeloid cells, neutrophils, and monocytes produce immune markers mediating interferon-stimulating gene transcription (IFI27, IFI35, ISG15, TXN., S100A4, S100A6, and FRP1) and pro-inflammatory cytokines, were highly detected. In contrast, the T cell titers (NK cells, T cells, mDCs, and pDCs cells) were lower than that in asymptomatic. Previous studies have reported detrimental effects of the IFN response, including its inflammatory role by recruiting more immune cells to the lungs to disrupt lung epithelial repair and the pulmonary epithelial barrier (7, 8) and suppressing pathway-related T cell functions (9) during severe COVID-19. Hence, this study by Xu et al. confirms that high IFN response (stimulated by high viral load (Nagaoka et al.) inhibits the maturation of naïve CD8^+^ T cells triggered during the early stage of the infection. More interestingly, the early strong IFN response impairing induction of CD8^+^ T-cells can lead to earlier fatality in patients with an already depleted titer of naïve CD8^+^ T cells (including elderly and immunosuppressed patients).

Aligning with before mentioned Xu et al.’s findings, Nagaoka et al. found that patients who later developed severe COVID-19 (characterized by hypoxemic respiratory failure) had high titer of INF-I response, specifically INF-α (but not IFN-β) and an increased level of pro-inflammatory cytokines (IL-6, and CXCL10) in the early stage of the infection. Moreover, transcriptomic study on lung samples from succumbed COVID-19 patients revealed higher levels of pulmonary IFN inducing genes than that of mild COVID-19 recovered patients (10) and which correlated with both cytokine storm and organ failure (sepsis) caused by necrosis, apoptosis, and pyroptosis, (Zhu et al. and reviewed by Moga et al.). Therefore, COVID-19 patients producing INF-α, IL-6, and CXCL10 at the early stage of the infection should be considered at high risk of respiratory failure and require urgent hospitalization to prevent fatalities.

While detecting IFN response at the early stage of COVID-19 infection is highly suggested to determine the potential severity of a beginning COVID-19 infection, Lai et al. revealed novel determining parameters/markers, which might also explain the severity of the infection, and, therefore, need to be monitored as well. Lai et al. confirmed that serum levels of autoantibodies against ACE2 are significantly higher in severe COVID-19 patients than in controls, and correlate with severity, suggesting that SARS-CoV-2 manage to induce anti-ACE2 antibodies leading to ACE2-specific autoimmune reaction-associated disease, possibly increasing sepsis and worsening the pathophysiology of the infection. Specifically, the amino acid residues P463, F464, E465, R466, D467, and E471 SARS-CoV-2 RBD are the primary residues recognized by the anti-ACE2-cross reactive RBD-specific antibodies. Notably, these residues are less or not reported as a variable in VOCs, suggesting that they are selective residues for the benefit of SARS-CoV-2, hence worsening pathological conditions. Besides, numerous currently available COVID-19 vaccines are developed from SARS-CoV-2 spike protein. Therefore, further studies must elucidate whether anti-ACE2-cross reactive RBD-specific antibodies are induced after SARS-CoV-2 vaccination and their potential pathological effects.

The continuous emergence of SARS-CoV-2 variants and their high ability to escape natural and vaccine-induced immunity, the reportedly life-threatening effects of respiratory bacterial coinfections, the resistance of COVID-19 to the currently repositioned treatment, and the lack of specific anti-COVID-19 treatment require more researches for safe and cost-effective alternatives to preventing COVID-19 disease.

The study by Smith et al. supports the fact that multi-pathogen coinfection with SARS-CoV-2 is a main infection pattern of increased pathological disorder. However, Cai et al. would like to draw attention to the contribution of coinfection with parasitic worms (non-pulmonary infections) in anti-COVID-19 strategies, from the hypothesis stating, “*Co-evolved microbes and other pathogens, including helminths, could help to establish appropriate immunomodulatory function and thus protect the host against a large spectrum of immune-related disorders*” (11). Hence, there is more evidence of a positive and attractive immunomodulatory response elicited by parasites, which may restore multi-system sepsis caused by other pathogens, including SARS-CoV-2. For instance, Cai et al. highlighted that chronic parasite infection induces an immunosuppressive and regulatory T-helper response that balances and lowers the inflammatory Th1/Th17 response triggered by SARS-CoV-2 infection in critically ill COVID-19 patients, restricting the severity of COVID-19 disease. Coinfection with helminths induces an anti-inflammatory Th-2 response characterized by the production of anti-inflammatory cytokines (IL-4, IL-5, and IL-13)-producing Th-2 cells. These anti-inflammatory cytokines could restore inflammatory-associated damage and thus sepsis caused by pro-inflammatory cytokines induced by activation of Th-1/Th-17 response. More precisely, these immunomodulatory responses are induced after the administration of helminth-derived products and attenuate the severity of sepsis, restoring covid-19-associated organ damages.

While Cai et al. could propose controlled helminth infection or using helminth products to control SARS-CoV-2 severity and reduce mortality, Santos et al. demonstrated an anti-SARS-CoV-2 protective activity of a carefully selected cocktail of cannabidiol and terpene, which may serve as natural extract therapeutic. In fact, the cannabinoid can boost immune response through activation of cannabinoid receptor 2 (CN2R) (12–14) and exhibit anti-inflammatory effect (15) in COVID-19 patients, while terpenes are known to enhance phytocannabinoid action; therefore, their combination was expected to synergistically protect against SARS-CoV-2, which is demonstrated in the study by Santos et al.

IgG Fc fragment plays important roles in viral clearance by activating the classical complement pathway and mediating infected cell clearance through binding to Fc gamma receptors (FcγRs), which activate antibody-dependent cellular cytotoxicity (ADCC) and antibody-dependent cellular phagocytosis (ADCP). In addition, since ACE2 is a known high-binding affinity receptor of a spike, designing ACE2-derived-Fc antibodies would be beneficial to boost effective COVID-19 immunity. In this regard, Wine and colleagues have engineered three ACE2-Fc (flACE2-Fc, EflACE2-Fc, and trACE2-Fc) by modifying ACE2 to enhance the existing binding affinity (neutralization) of ACE2 to SARS-CoV-2 S protein. The proposed high binder spike-specific ACE2-Fc demonstrated distinct but promising enhanced neutralization effects against SARS-CoV-2 and increased Fc-associated effector functions against virus-infected cells. In addition, despite the exciting results by Bahnan et al. demonstrating that non-neutralizing antibodies could be used as they can confer protection against SARS-CoV-2 through Fc-mediating phagocytosis, high-binding affinity-associated neutralizing antibodies (such as engineered ACE2-Fcs) constitute a safer way against SARS-CoV-2 infection, because neutralizing antibodies are multi-effective, whereas reported antibody-dependent enhancement (ADE) disorders are related to non-neutralizing antibodies (16).

Overall, this Research Topic compiled studies that brought novelties in aspects of SARS-CoV-2–host interactions and advanced our knowledge of the immunological mechanisms underlying COVID-19 severity. Moreover, these studies present alternative solutions for effective prophylactic and therapeutic interventions, which are essential for our preparedness to control the current pandemic state and for future emergence and re-emergence of more pathogenic SARS-CoV-2 variants or similar viruses.

## Author contributions

TJ provided funding, conceptualized the idea of the topic, and edited the manuscript. JK conceived the topic and drafted and edited the manuscript. All authors contributed to the article and approved the submitted version.
